# DMF-Net: a deep multi-level semantic fusion network for high-resolution chest CT and X-ray image de-noising

**DOI:** 10.1186/s12880-023-01108-0

**Published:** 2023-10-09

**Authors:** Tapan Kumar Nayak, Chandra Sekhara Rao Annavarappu, Soumya Ranjan Nayak, Berihun Molla Gedefaw

**Affiliations:** 1 Department of CSE, IIT(ISM) Dhanbad, Sardar Patel Nagar, Dhanbad, 826004 Jharkhand India; 2 School of Computer Engineering, KIIT Deemed to be University, Bhubaneswar, 751024 Odisha India; 3 Department of Health Informatics, Arba Minch University College of Medicine and Health Science, Arba Minch, Ethiopia

**Keywords:** Cascaded Feature, DMF-Net, CT, X-Ray

## Abstract

Medical images such as CT and X-ray have been widely used for the detection of several chest infections and lung diseases. However, these images are susceptible to different types of noise, and it is hard to remove these noises due to their complex distribution. The presence of such noise significantly deteriorates the quality of the images and significantly affects the diagnosis performance. Hence, the design of an effective de-noising technique is highly essential to remove the noise from chest CT and X-ray images prior to further processing. Deep learning methods, mainly, CNN have shown tremendous progress on de-noising tasks. However, existing CNN based models estimate the noise from the final layers, which may not carry adequate details of the image. To tackle this issue, in this paper a deep multi-level semantic fusion network is proposed, called DMF-Net for the removal of noise from chest CT and X-ray images. The DMF-Net mainly comprises of a dilated convolutional feature extraction block, a cascaded feature learning block (CFLB) and a noise fusion block (NFB) followed by a prominent feature extraction block. The CFLB cascades the features from different levels (convolutional layers) which are later fed to NFB to attain correct noise prediction. Finally, the Prominent Feature Extraction Block(PFEB) produces the clean image. To validate the proposed de-noising technique, a separate and a mixed dataset containing high-resolution CT and X-ray images with specific and blind noise are used. Experimental results indicate the effectiveness of the DMF-Net compared to other state-of-the-art methods in the context of peak signal-to-noise ratio (PSNR) and structural similarity measurement (SSIM) while drastically cutting down on the processing power needed.

## Introduction

X-ray and Computed Tomography (CT) are two distinct imaging techniques used frequently in the medical field for corporal inspection of human lungs. An X-ray is a commonly used and widely available imaging technique, while a CT scan is similar to MRI in that it produces high-quality body organ images. Both types of scans produce images in different ways. The CT scan takes pictures of body organs from all angles, enhancing its accuracy, while X-rays use electromagnetic waves to flow through the patient’s body, producing black-and-white photos of the inside structure. In radiology, CT scans and X-rays are frequently used for diagnostic purposes.

The quality of CT and X-ray pictures has deteriorated for a variety of causes, including the following:

Blurred image: These medical pictures are blurred due to incorrect protocol parameters and patient movement [1]. Patient movement is caused by various variables, including an increase in heart rate, respiration, fluctuations in the number of pixels while scanning in a uniform material, and the patient’s unwillingness to cooperate. Blurring rises in direct proportion to the degree of movement.

Field of View (FOV): When the resolution is changed to take a picture of a smaller or larger area, the image quality also gets worse.

Artefact: Artifacts are incidental, supplementary graphics that appear alongside the main image, like metal artifacts, beam hardening, and faulty equipment, accidental damage, or inappropriate input can result in artefacts.

It has a large impact on reducing the model’s accuracy during classification, detection, segmentation, and registration when the picture quality is influenced by various amounts of noise [2–4]. As a result, it is important to make a model for removing noise from medical images like CT and X-rays as part of the pre-processing [5, 6].

To denoise the images various models are used can be categorized in two types (1) traditional model and (2) deep models.

(1) Traditional models: Different traditional methods used are linear smoothing, median filtering, wiener filtering, anisotropic diffusion, and wavelet-based methods.

i. Linear smoothing: Here a noisy image y convolved with a Gaussian filter k, to clean up an image.
  1 $$\begin{aligned} \hat{x}=y \times k \end{aligned}$$
  It can also be done in a fourier domain as follows:
  2 $$\begin{aligned} \hat{X}=Y \odot K \end{aligned}$$
  Here, capital letters stand for the Fourier transform of their counterparts (for example, Y = F(y), where F is the Fourier transform), and $\odot$ stands for the element-wise product. In the Fourier domain, K is also gaussian.
ii. Median Filtering: Median filtering can be used instead of linear smoothing. The idea behind median filtering is to take an image and work on it one pixel at a time. Also, every pixel is supplemented by the value that is in the middle of a group of pixels that are close to it. So, the method can also be seen as a way to filter, although the filter is not linear.
iii. Wiener Filter: Linear prediction, signal restoration, and channel equalization are just a few examples of the many uses for Wiener filters. This technique works for both additive noise and multiplicative noise.
iv. Anisotropic diffusion: Anisotropic diffusion, an iterative method that uses smoothing, can be used to remove noise from images. This strategy makes an effort to meet the following conditions: (a) Object borders must be kept intact, and (b) noise must be effectively filtered out in areas of high similarity. The approach is so-called because its mathematical underpinnings are similar to those of heat diffusion equations and because its smoothing or diffusion is applied in discrete regions rather than globally.
v. Wavelet-based method: Here, an image is converted into a wavelet domain and wavelet coefficients. Then, the inverse wavelet transform is used to get the denoised image.

(2) Deep models: Deep learning was first introduced for image de-noising in 1989 by Chiang and Sullivan. Here the proposed neural network uses blur function and additive noise to get a clean image. The network then used weight values to get rid of complicated noise [7]. To cut down on the high cost of computation, a feedforward network had been suggested to achieve a tradeoff among both de-noising performance and efficiency [8]. After that, further optimization techniques were employed to speed up the convergence of the network and improve the performance of de-noising [9]. Also by raising the depth or modifying the activation function, novel network designs shown competitive in removing noise [10]. But these models require parameters to be set manually and it got resolved with gradient descent [11, 12]. Because of the aforementioned reasons, convolutional neural networks (CNNs) were proposed with vanishing gradients and different activation functions such as sigmoid [13] and tanh [14] but it needs a computationally effective platform to implement. As a result the ImageNet challenge started in 2012 brings different pre-trained models like AlexNet, VGGNet, MobileNet [15–17] etc. to deal with. These models were used for de-noising image starting from 2015 [18, 19]. Image de-noising methods mostly use the mathematical model y = x + $\eta$ to get all the clean images, x, where $\eta$ and y are just additive noise with standard deviation($\sigma$) and a noisy image, respectively. Many publications used this formulation as the bedrock for their models [20] in the past. Zhang et al. [21] devised a deep convolutional neural network entitled De-noising CNN with Batch Normalization (BN) and Residual Learning (RL) to reconstruct the clean image. Autoencoders and stacked sparse autoencoders have been applied to enhance image de-noising performance with higher efficiency for spatial correlations [22]. The information from the final layer got incorporated in most CNN-based models, while low-level information remained overlooked. Although the previous methods are more appealing toward image restoration, they have distinguished drawbacks: (1) The dense network [23] does not use the shallow or hidden layers effectively. (2) Most of the methods trade-off with complex background which hides required features.

Therefore the proposed DMF-Net is composed of Dilated Feature Extraction Block (DFEB), Cascaded Feature Block(CFB), and Prominent Feature Refinement Block(PFRB) trained with decaying the learning rate. The contributions of this work are as follows,

- The proposed DMF-Net contains dilated convolution and batch normalization is used with a unique idea of combined feature matrix from different layers.
- The proposed model is trained and evaluated with CT and X-ray images separately and combined.
- The proposed model addresses the high-level abstraction of radiographs, hence eliminating the requirement of a handcrafted feature extraction process.
- The recent research trends often give importance to other layers for feature extraction not only the last layer, as it diversifies the image features. Getting inspired by this literature, an effort has been made with other layers.

## Related work

Different CNN models have been designed for object detection and retrieval of the clear image with modified preexisting networks or designing networks with different plug-ins to improve the model results [24, 25]. Most of the CNNs has been designed with the aim of improving efficiency and accuracy w.r.t. de-noising. Dabov et al. [20] proposed a model with enhanced sparcity to convert 2D array to 3d array. They got a significant improvement with specially designed weiner filter. Zhang et al. [26] proposed weighted nuclear norm minimization(wnnm) technique to take the advantage of nonlocal self similarity. Buades et al. [27] put emphasis on nonlocal means(nlm) to focus on structual preservation of image. Portilla et al. [28] used statistical model with bayesian estimator to eliminate gaussian noise. Chen et al. [29] proposed residual encoder with decoder convolutional network(RED-CNN) by patch based training for CT-images. Gondara et al. [30] developed an autoencoder with convolutional layers to deal with heterogenious data to address less computational complexity. Kang et al. [22]designed an algorithm with directional wavelet to address the photon starvation in CT-images. Zheng et al. [31] enhanced the spatial adaptivity of nlm with element-wise fractal dimension. Duan et al. [32] proposed a new second-order total generalised variation (TGV) decay model to get rid of speckle noise. Yang et al. [33] developed tensor based adaptive control for principal component analysis with a searching window for image de-noising. Zohair et al. [34] proposed a phase preserving approach got better peak signal noise ratio(psnr). Trinh et al. [35] formulated quadratic programming on weighted image block resulting better performance. Chen et al. [36] introduced deep boosting framework integrating with various cnn to generate required features for noise removal. Also the increased training samples has shown better performance in suppressing the noise. As experimented with a generative adversarial network (GAN) [37] a discriminative network has been used to increase the samples for the training purposes.

## Methodology

This section contains an extensive description of the DMF-Net. The noisy X-ray and CT scans are developed by adding a particular noise value with the clean image, which has been described as Y = X + $\eta$, used as a souce of data to DICN, where X : clean image, Y: input noisy image with $\eta$ : external noise. The basic goal is to get the DICN to learn with a noisy image and then replicate a clean or noise-free image. The main aim of the model is to make it learn the noisy data to predict the clean image.

### Feature dialation

The architecture with dilated convolutions [38] does the expansion of the receptive field, which increases accuracy with no resolution loss. Integrating multi-scale information with sub-sampling in image classification models reduces resolution. It helps in the area expansion of image data without pooling. The main aim is to cover wider pixel information to convolve for the output feature with the same cost of computation. Here the dilation factor(d) determines what will be the result by convolving with different values of d. The same kernel parameter can be used to retrieve more information. The value of d=1 means the kernel gets mapped with the same size of input but from d=2 onwards one pixel gets skipped while mapping with input.

**Fig. 1:**
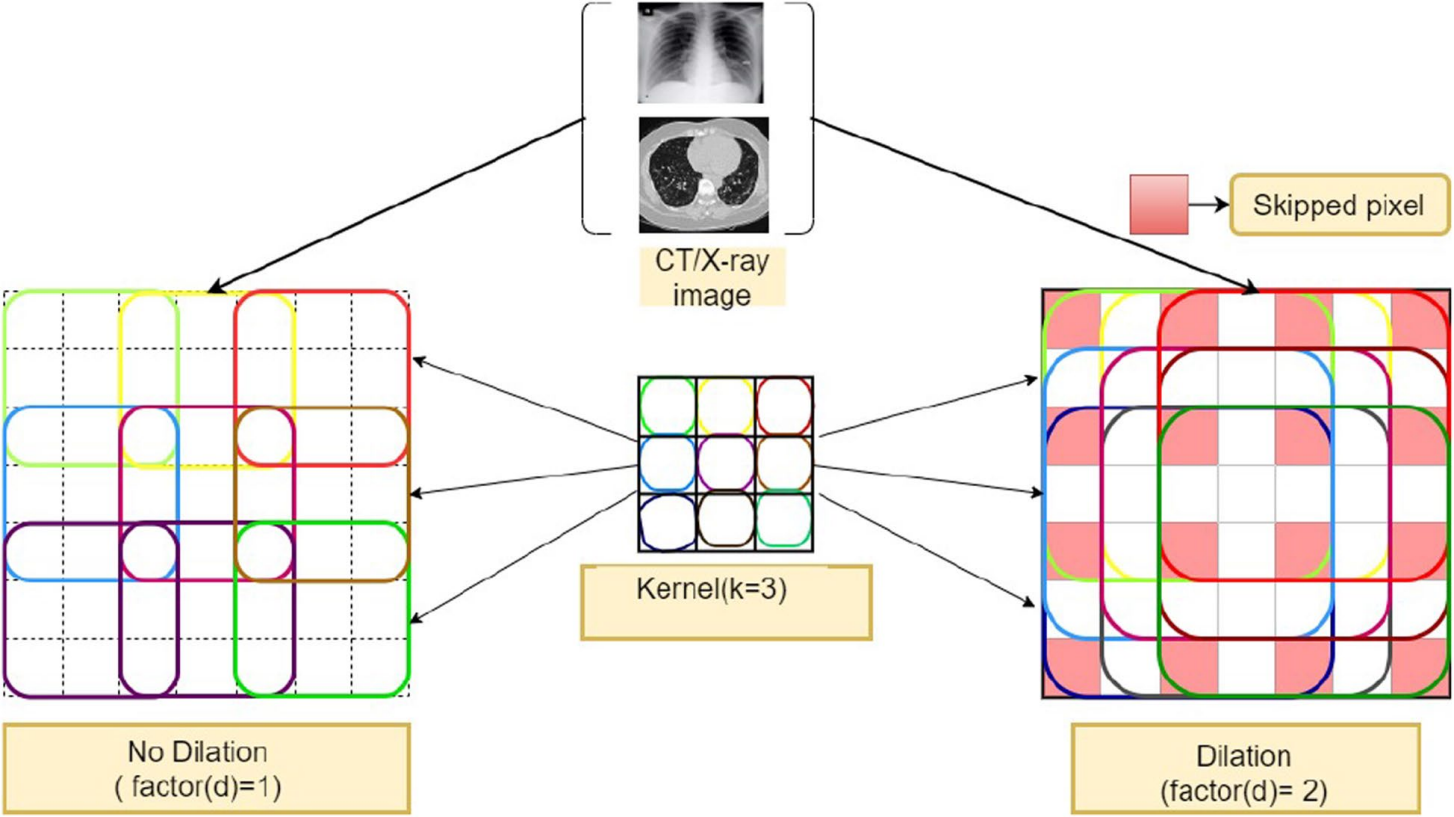
Convolution with(d=2) and without(d=1) dilation

For a dilated convolution or a d-dilated convolution named *d is used. The familiar discrete convolution is simply the 1-dilated convolution. Let $f_0$, $f_1$, . . . , $f_n$ : Z $\rightarrow$ R be the functions and $k_0$, $k_1$, . . . , $k_n$ : $\Omega \rightarrow$ R be the $3\times 3$ filters. Kernel filters has been applied with increasing dilation:

The convolution operation is performed as defined (F $*$ k)(p) = X

3 $$\begin{aligned} (F*k)(p) = \sum \limits _{m+t=p} F(m)k(t) \end{aligned}$$

For the dilated convolution operation, let d is the dilation factor then (F $*_d$ k)(p) = X

4 $$\begin{aligned} (F*_dk)(p) = \sum \limits _{m+dt=p} F(m)k(t) \end{aligned}$$

Here $*_d$ is assumed as the dilation factor. During convolution operation, the dilation factor determines how many pixels will be skipped with d dilation, d-1 no. of pixels skipped from input receptive(p) during convolution performed with the kernel. The Fig. 1 shows how the convolution performed with the skipping of pixels with different dilation factor.

###  Proposed DMF-Net architecture

**Fig. 2:**
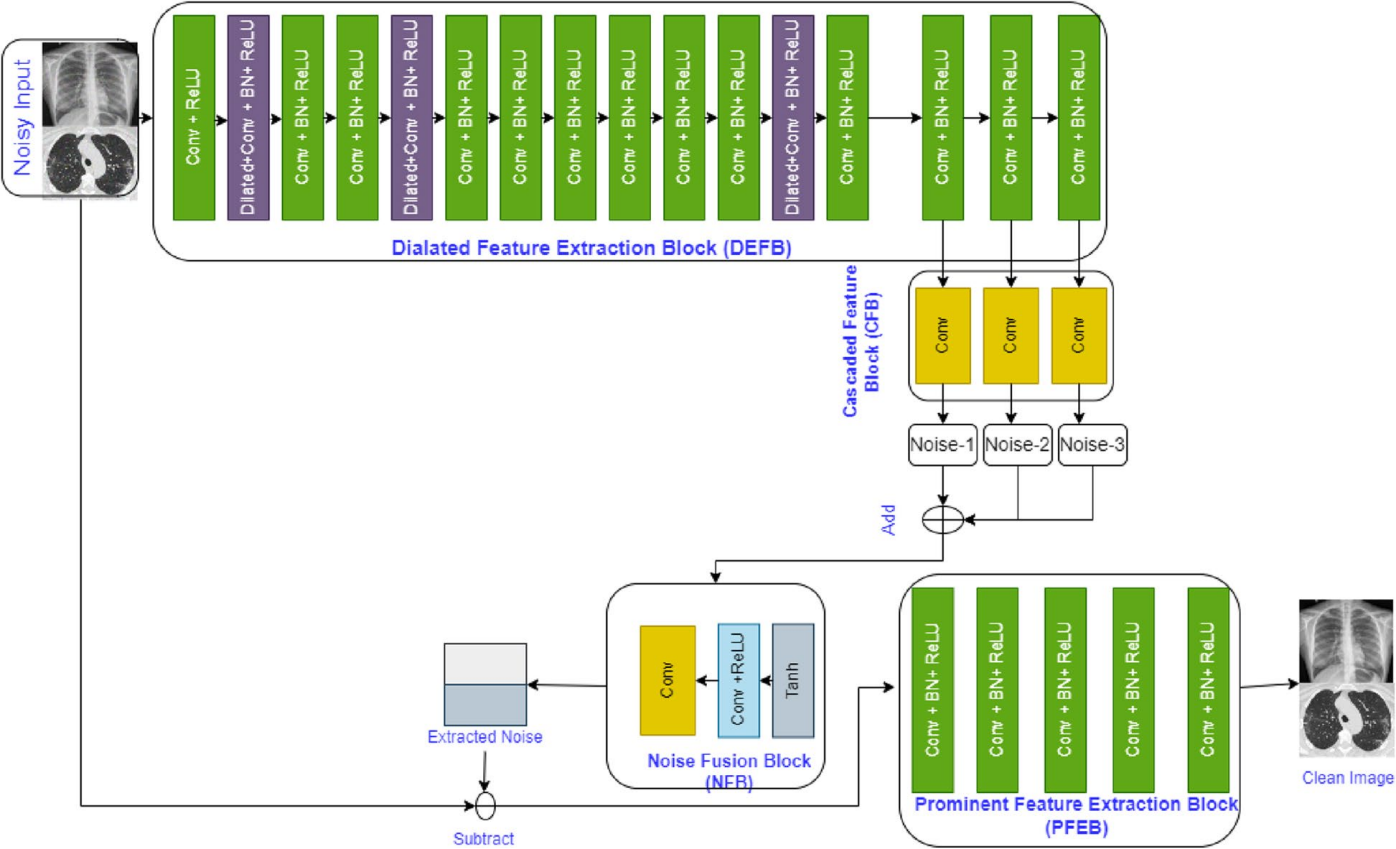
Proposed model DMF-Net with different blocks

The proposed architecture DMF-Net in Fig. 2 with the layer description in Table 1, contains three parts, DFEB, CFB and PFRB. The noisy images are the input to the DFEB box which has sub-modules like convolution, convolution with batch normalization and dilated convolution with batch normalization. CFB block does the inner layer cascading by adding the output features from low, mid and high levels. Its aim is to give all the levels equal chances for the extraction of the noisy feature. These get concatenated to enhance the feature responsible for the noise. DMF-Net is designed with total of 26 convolution layers. The Dilated Feature Extraction Block consist of dilated 3 $\times$ 3 with ReLU [39] activation operation and normalising of several batches (BN) [40] and operation of ReLU activation (blue box).

It adds non-linearity to the network. The Batch Normalization performed in different layers enhances the training with an improved learning rate. Here from layer numbers 14th, 15th and 16th, the feature has been taken with convolution. This has been done keeping in mind that the features from other layers may enhance the noisy information. The three features from these layers are concatenated and passed through a noise fusion block(NFB). The NFB contains Tan hyperbolic function with convolution and ReLU. Finally, it gets convolved to give the final net extracted noise. The NFB module refines the fused noise for the final considered net noise feature (NNF). This NNF is subtracted from the noisy image to give a clear image after passing through a prominent feature extraction block(PFRB). It contains four modules with one convolution with ReLU and three convolutions with Batch Normalization and ReLU. The PFRB block enhances the image quality by considering the prominent features. Here throughout the model same kernel size has been used i.e. $3\times 3$ kernel. The final image size and the input image size is same. The $128\times 128$ image size has been considered throughout the experiment by keeping the stride and padding one in every convolution layer. In order to extract more complete features and thus improve the model potential.

**Table 1:** Detailed description of layers in DMF-Net

Operation Layer		No of Filters	Size of Each Filters	Dilation	Stride Value	Padding Value	Size of Output Image
Input Image		-	-	-	-	-	$128 \times 128 \times 1$
Convolution Layer	Convolution	64	$3\times 3\times 1$	1	$1\times 1$	$1\times 1$	$128 \times 128 \times 64$
(one)	ReLU	-	-	-	-	-	$128 \times 128 \times 64$
Convolution Layer	Convolution+BN	64	$3\times 3\times 1$	2	$1\times 1$	$1\times 1$	$128 \times 128 \times 64$
(one)	ReLU	-	-	-	-	-	$128 \times 128 \times 64$
Convolution Layer	Convolution+BN	64	$3\times 3\times 1$	1	$1\times 1$	$1\times 1$	$128 \times 128 \times 64$
(two)	ReLU	-	-	-	-	-	$128 \times 128 \times 64$
Convolution Layer	Convolution+BN	64	$3\times 3\times 1$	2	$1\times 1$	$1\times 1$	$128 \times 128 \times 64$
(one)	ReLU	-	-	-	-	-	$128 \times 128 \times 64$
Convolution Layer	Convolution+BN	64	$3\times 3\times 1$	1	$1\times 1$	$1\times 1$	$128 \times 128 \times 64$
(six)	ReLU	-	-	-	-	-	$128 \times 128 \times 64$
Convolution Layer	Convolution+BN	64	$3\times 3\times 1$	2	$1\times 1$	$1\times 1$	$128 \times 128 \times 64$
(one)	ReLU	-	-	-	-	-	$128 \times 128 \times 64$
Convolution Layer	Convolution+BN	64	$3\times 3\times 1$	1	$1\times 1$	$1\times 1$	$128 \times 128 \times 64$
(four)	ReLU	-	-	-	-	-	$128 \times 128 \times 64$
Convolution Layer	Convolution	1	$3\times 3\times 1$	1	$1\times 1$	$1\times 1$	$128 \times 128 \times 1$
(three)	-	-	-	-	-	-	$128 \times 128 \times 1$
	Tanh	-	-	-	-	-	$128 \times 128 \times 1$
Convolution Layer	Convolution	1	$3\times 3\times 1$	1	$1\times 1$	$1\times 1$	$128 \times 128 \times 1$
(one)	ReLU	-	-	-	-	-	$128 \times 128 \times 1$
Convolution Layer	Convolution+BN	1	$3\times 3\times 1$	1	$1\times 1$	$1\times 1$	$128 \times 128 \times 1$
(one)	ReLU	-	-	-	-	-	$128 \times 128 \times 1$
Convolution Layer	Convolution+BN	64	$3\times 3\times 1$	1	$1\times 1$	$1\times 1$	$128 \times 128 \times 64$
(one)	ReLU	-	-	-	-	-	$128 \times 128 \times 64$
Convolution Layer	Convolution+BN	64	$3\times 3\times 1$	1	$1\times 1$	$1\times 1$	$128 \times 128 \times 64$
(three)	ReLU	-	-	-	-	-	$128 \times 128 \times 64$
Convolution Layer	Convolution+BN	1	$3\times 3\times 1$	1	$1\times 1$	$1\times 1$	$128 \times 128 \times 1$
(one)	ReLU	-	-	-	-	-	$128 \times 128 \times 1$

###  Training strategy

The mixed data training approach for deep network construction has been proposed here. The data set contains both CT and X-ray image data for the training. The basic goal of mixed training is for the model to learn a number of desirable properties. The features change drastically when a clinical image is added with a certain type of noise. Because of this diversity in noisy images, it has been experimented with here to train the model with mixed clinical images. The results found here are also promising. Also, the DMF-Net is trained separately with CT images, augmented CT images, X-ray images, and augmented X-ray images with normally distributed specific noise levels (15, 20, 25, respectively) and blind noise. The model is trained upto 60 epochs with dynamic learning rate $(\alpha$=0.001, 0.0001, 0.00001, 0.000001)has been considered for the smooth convergence. Learning rate ($\alpha$ = 0.001 (upto 20 epoch), 0.0001 (20 <epoch $\ge$ 40), 0.00001 (40< epoch $\ge$50) and 0.000001 (50< epoch$\ge$60). The contribution of low, mid, and high-level aspects was properly considered by integrating the features from the last three layers to determine noise.

Features from the last three-layer have been added to find the net feature f(L).

5 $$\begin{aligned} f_{DMF-Net}(L)= f_{DMF-Net}(L_{17}) + f_{DMF-Net}(L_{18}) + f_{DMF-Net}(L_{19}) \end{aligned}$$

where $L_{17}, L_{18} ,L_{19}$ are layer 17, layer 18 and layer 19. The final feature after convolution is considered as the final noise feature $f_{DMF-Net}(N)$. The predicted denoised image($I_{P}$) has been determined between the input noisy image($I_{noisy}$) and f(N).

6 $$\begin{aligned} I_{P}= I_{noisy}-f_{DMF-Net}(N) \end{aligned}$$

7 $$\begin{aligned} Loss(L)= \left\| I-I_{P}\right\| ^{2} \end{aligned}$$

Finally, the difference between the original noise-free picture (I) and the anticipated noise-free image is computed ($I_{P}$). After the loss is calculated, the optimization is done using adam optimizer [41] which is considered during training. The number of parameters get optimized due to the noise features extracted during training. However, the dynamic learning rate with respect to different epochs helps in faster convergence. Because the loss calculated in gradient gets transmitted due to the learning function, reflecting the variation between the original noise and predicted noise with the parameter.

## Experimental results

### Details about the data as well as the implementation

Here our data folder contains CT image files as well as X-Ray image files. CT files of counts 1647 are collected from kaggle.com [42, 43] and GitHub.com [44], each one of grayscale lung CT slices of size $128\times 128$. The data folder is again divided in two folders as CT-training and CT-testing. Randomly the files are chosen from the training folder containing 1352 files with a ratio of 90:10 as training : validation and 295 files for testing. 1550 lungs X-Ray files are collected from kaggle.com [45] and distributed in two folders as X-Ray-training with 1292 files and X-Ray-testing with 258 files. Files from X-Ray-training folder are again chosen randomly as training : validation with ratio 90:10.

Our deep model DMF-Net is developed in PyTorch. Here Adam optimizer is set for training the network with starting learning rate of 0.001 and it is scaled down by 10 after 20, 40 and 50 epochs. Training is done with 60 epochs with a batch size of 32. After training the network, the states of the model for 60 epochs have been saved with 60 .pth files. The system set up for the training and testing is NVIDIA-SMI 460.32.03 and CUDA version 11.2 with Tesla T4 and 32 GB RAM.

### Evaluation metrics

The peak signal to noise ratio (PSNR) and the structural similarity index (SSIM) are employed as assessment criteria in this study. The mathematical formulation of PSNR is

8 $$\begin{aligned} PSNR = 20\log _{10}\frac{MAX_{f}}{(MSE)^{\frac{1}{2}}} \end{aligned}$$

where, peak signal to noise ratio (PSNR) is the ratio of the maximum pixel value of a noise-free image to the maximum pixel value of the image. and $(MSE)^{\frac{1}{2}}$ is the root mean square error.

9 $$\begin{aligned} MSE = \frac{1}{pq}\sum \limits _{0}^{p-1}\sum \limits _{0}^{q-1}\left\| I(i,j)-I'(i,j)\right\| ^{2} \end{aligned}$$

where I(i,j) is the pixel data of our noise free image, $I'(i,j)$ represents the pixel data of denoised image predicted by model. p: total rows of image data pixels and q: total columns of image data pixels. SSIM measures the incessant difference between similar images. It never conclude about the original or denoised image.

10 $$\begin{aligned} SSIM(I,I')=\frac{(2\mu _{I}\mu _{I'}+c_{1})(2\sigma _{II'}+c_{2})}{(\mu _{I}^{2}+\mu _{I'}^{2}+c_{1})(\sigma _{I}^{2}+\sigma _{I'}^{2}+c_{2})} \end{aligned}$$

where $\mu _{I}$ and $\mu _{I'}$ are mean of image I and $I'$, $\sigma _{I}$ and $\sigma _{I'}$ are standard deviation(s.d.) of image I and $I'$ and $\sigma _{II'}$ is the covariance of I and $I'$ with $c_{1} c_{2}$ as constants.

To observe the performance, DMF-Net is trained with specific noise and blind noise. Specific noise and blind noise has been normalized with noise level $N_{sp}$(15, 20, 25) and $N_{bl}$(0 to 55) for creating the noisy X-Ray as well as CT image. The table 2 shows the PSNR and SSIM resulted by training the model with CT image, X-Ray image and mixed images separately.

### De-noising CT image where DMF-Net trained with CT image

**Fig. 3:**
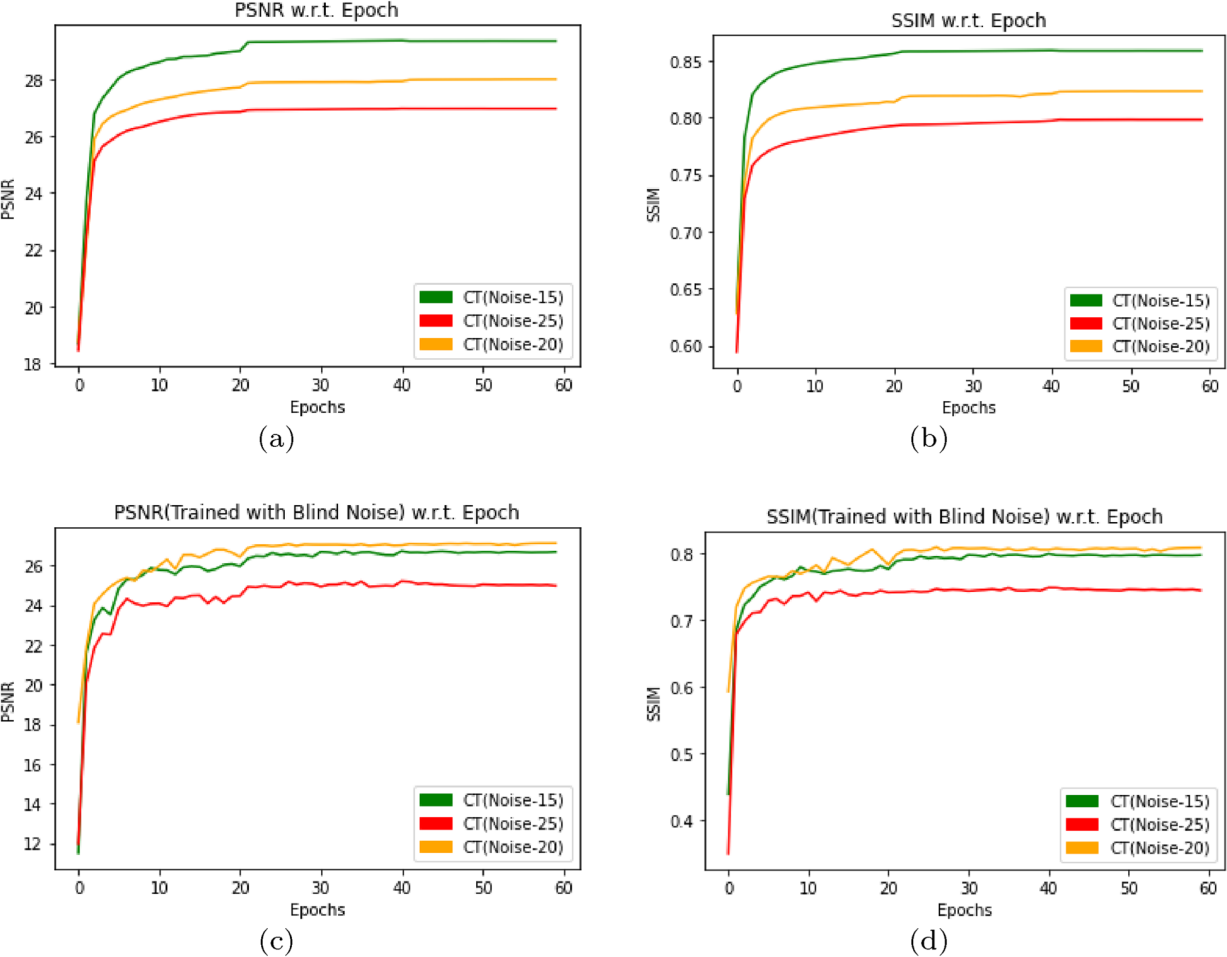
PSNR and SSIM w.r.t. Epoch by using DMF-Net without augmentation and with specific noise in (**a**, **b**) and blind noise in (**c**, **d**) for noise level($\sigma$) 15,20 and 25

**Fig. 4:**
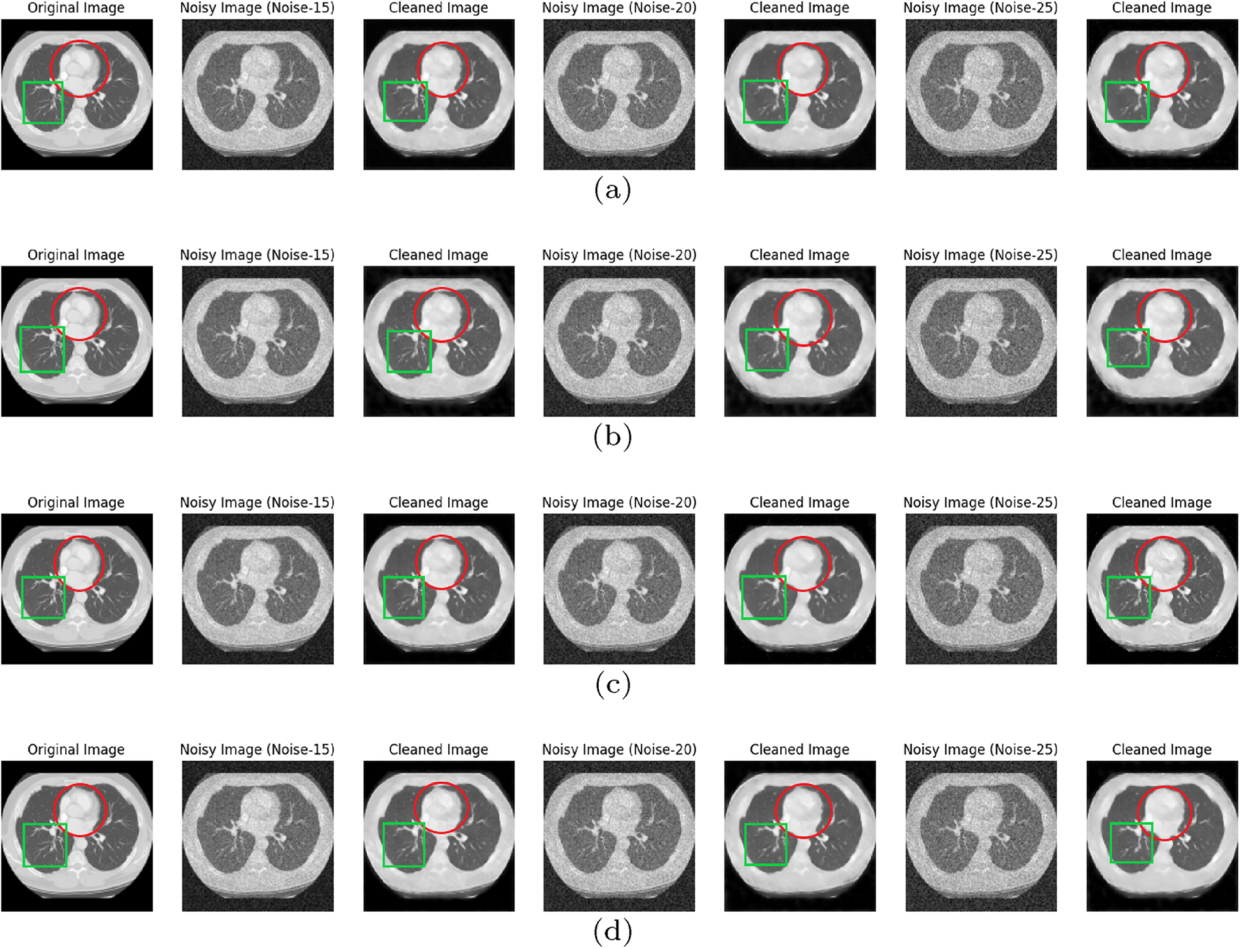
Original CT image with noisy($N_{sp}$=15,20,25 and $N_{bl}$=0 to 55) and denoised or cleaned image by DMF-Net where (**a** and **c**) shows for specific noise without and with augmentation in (**b** and **d**) shows for blind noise without and with augmentation; red circle and green box to observe inter lungs area and left lungs respectively with original image

In this section, DMF-Net trained with $128\times 128$ CT image files in the CT-training folder containing 1352 files with specific noise($N_{sp}$) and blind noise($N_{bl}$). The states of the model is saved for 60 epochs for evaluating the model with PSNR and SSIM. Again the model gets trained with augmented CT images created by flipping the image right-left and up-down with angle 90$^{\circ }$,180$^{\circ }$ and 270$^{\circ }$ randomly, generating 2458 image files. Also the states of the model for 60 epochs has been saved for evaluating the model with PSNR and SSIM. Without augmentation the trained model for a test data of 295 images achieved average PSNR and SSIM for $N_{sp}$=15,20 and 25 with [(26.66,0.7973),(27.09,0.8082) and (24.96, 0.7444)] respectively and for $N_{bl}$=0 to 55 with [(26.66,0.7973),(27.10, 0.8082),(24.96, 0.7444)] respectively as shown in Fig. 3(a) and (b). With augmentation the model after 60 epochs results average PSNR and SSIM for $N_{sp}$=15,20 and 25 with [(28.23,0.8294),(27.11, 0.7934) and (26.57,0.7787)] respectively and for $N_{bl}$=0 to 55 with [(28.23,0.8294),(27.11, 0.7934), (26.57,0.7787)] respectively as shown in Fig. 3(c) and (d). The plot for PSNR and SSIM w.r.t. epochs are described in Fig. 3 with different noise values. The resulted denoised image has been displayed in Fig. 4 for different noise type and the peculiar areas has been selected to compare with original image.

### De-noising X-Ray image where DMF-Net trained with X-Ray image

**Fig. 5:**
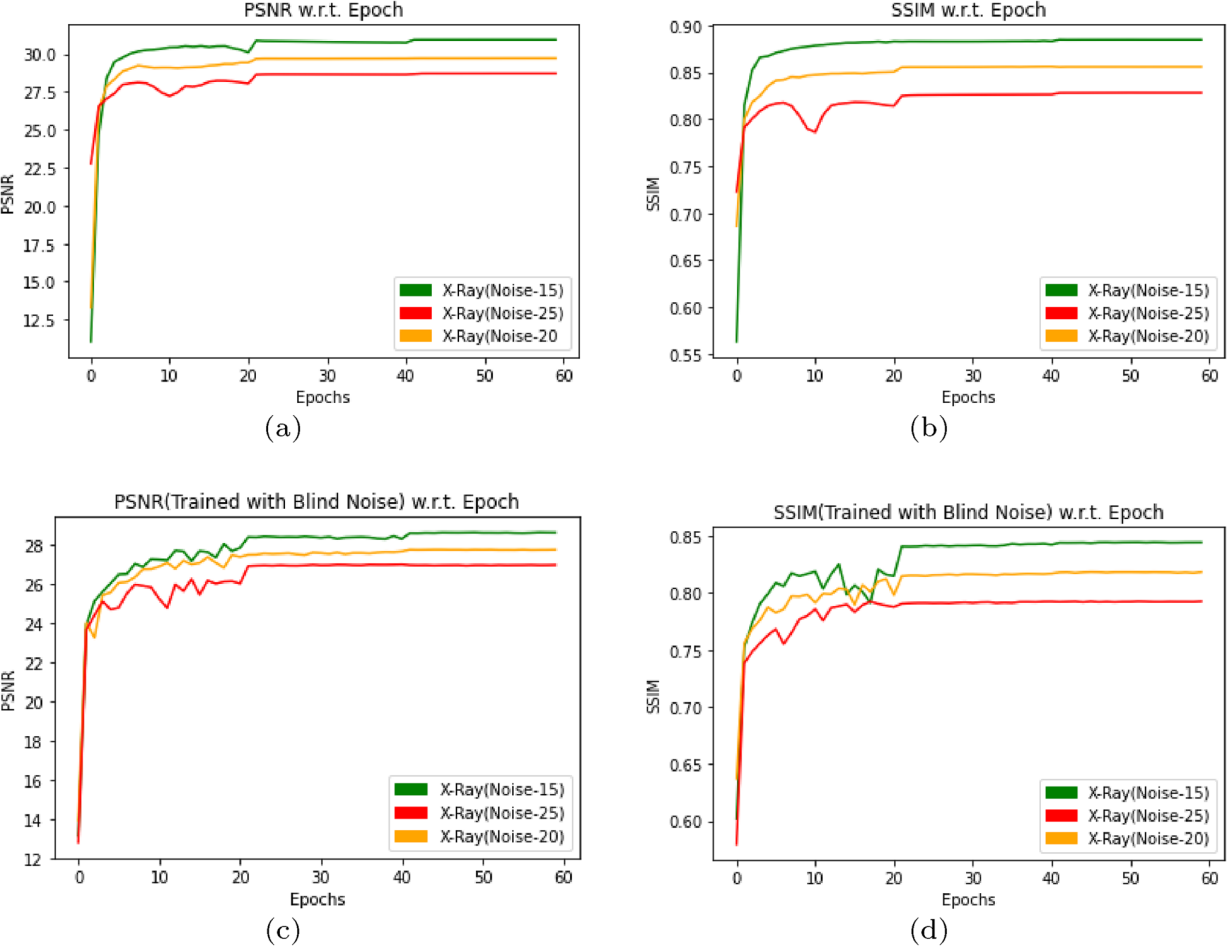
PSNR and SSIM w.r.t. Epoch by using DMF-Net without augmentation and with specific noise in (**a**, **b**) and blind noise in (**c**, **d**) for noise level 15,20 and 25

**Fig. 6:**
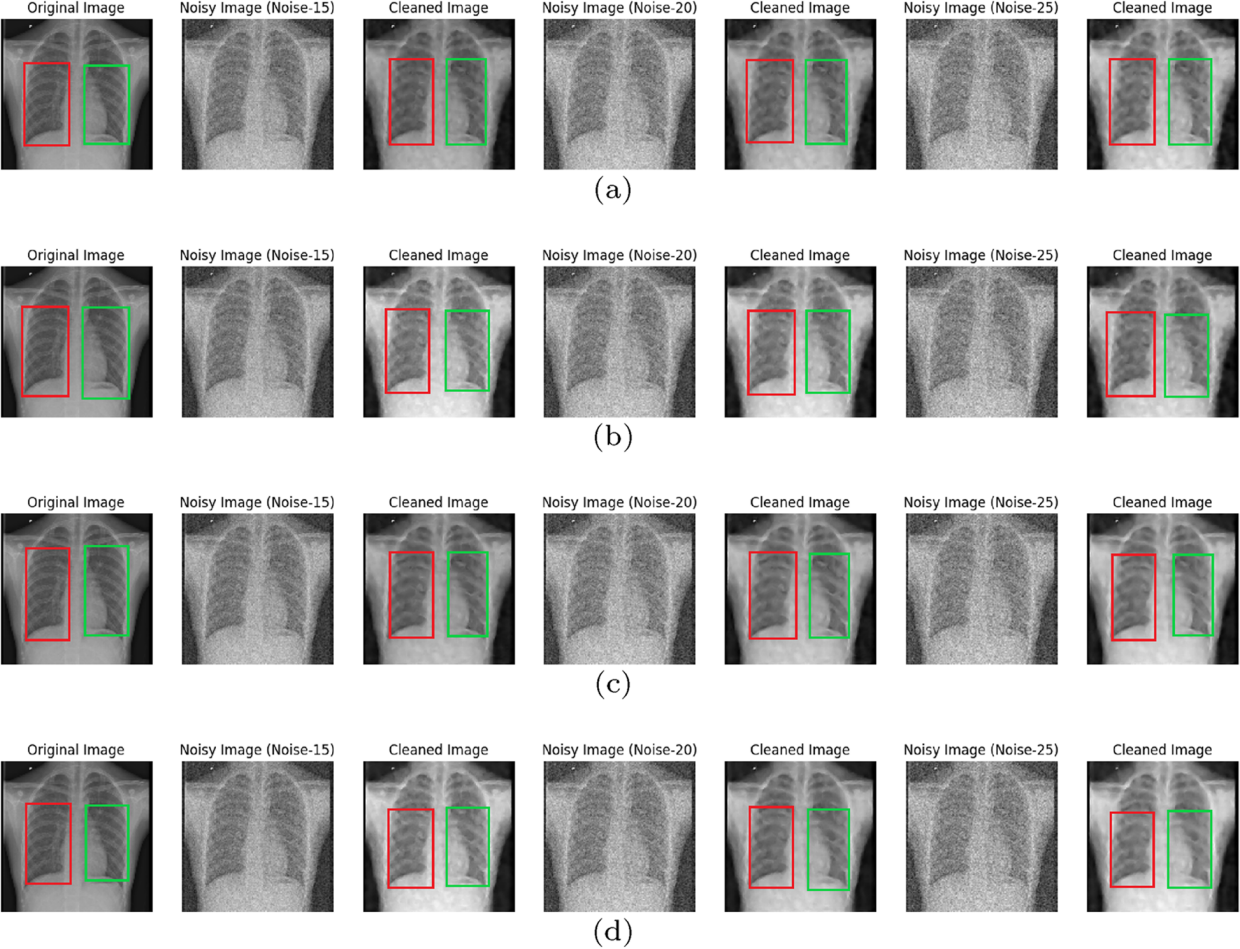
Original X-Ray image with noisy($N_{sp}$=15,20,25 and $N_{bl}$=0 to 55) and denoised or cleaned image by DMF-Net where (**a** and **c**) shows for specific noise without and with augmentation in (**b** and **d**) shows for blind noise without and with augmentation; red box and green box to observe left lungs and right lungs area respectively with original image

In this section, DMF-Net trained with $128\times 128$ X-Ray image files in X-Ray-training folder containing 1292 files with specific($N_{sp}$) and blind($N_{bl}$) noise observing PSNR and SSIM. Also the model performance is observed for augmented images generated by flipping right-left, up-down with angle 90$^{\circ }$,180$^{\circ }$ and 270$^{\circ }$ randomly, generating 2348 image files. The model is trained for 60 epochs. The trained model for a test data of 258 images achieved average PSNR and SSIM for $N_{sp}$=15,20 and 25 with [(30.94, 0.8848), (29.72,0.8560) and (28.72,0.8282)] respectively and for $N_{bl}$=0 to 55 with [(28.59,0.8445), (27.72, 0.8185),(26.94, 0.7926)] respectively as shown in Fig. 5(a) and (b). But with augmentation the model after 60 epochs results average PSNR and SSIM for $N_{sp}$=15,20 and 25 with [(31.03,0.8896), (29.95, 0.8637) and (29.19,0.8442)] respectively and for $N_{bl}$=0 to 55 with [(28.92,0.8525),(28.01, 0.8240),(27.19, 7954)] respectively as shown in Fig. 5(c) and (d) and some resulted images are shown in Fig. 6 for the visual comparison of some peculiar areas.

### De-noising both CT and X-Ray image where DMF-Net trained with mixed image

**Fig. 7:**
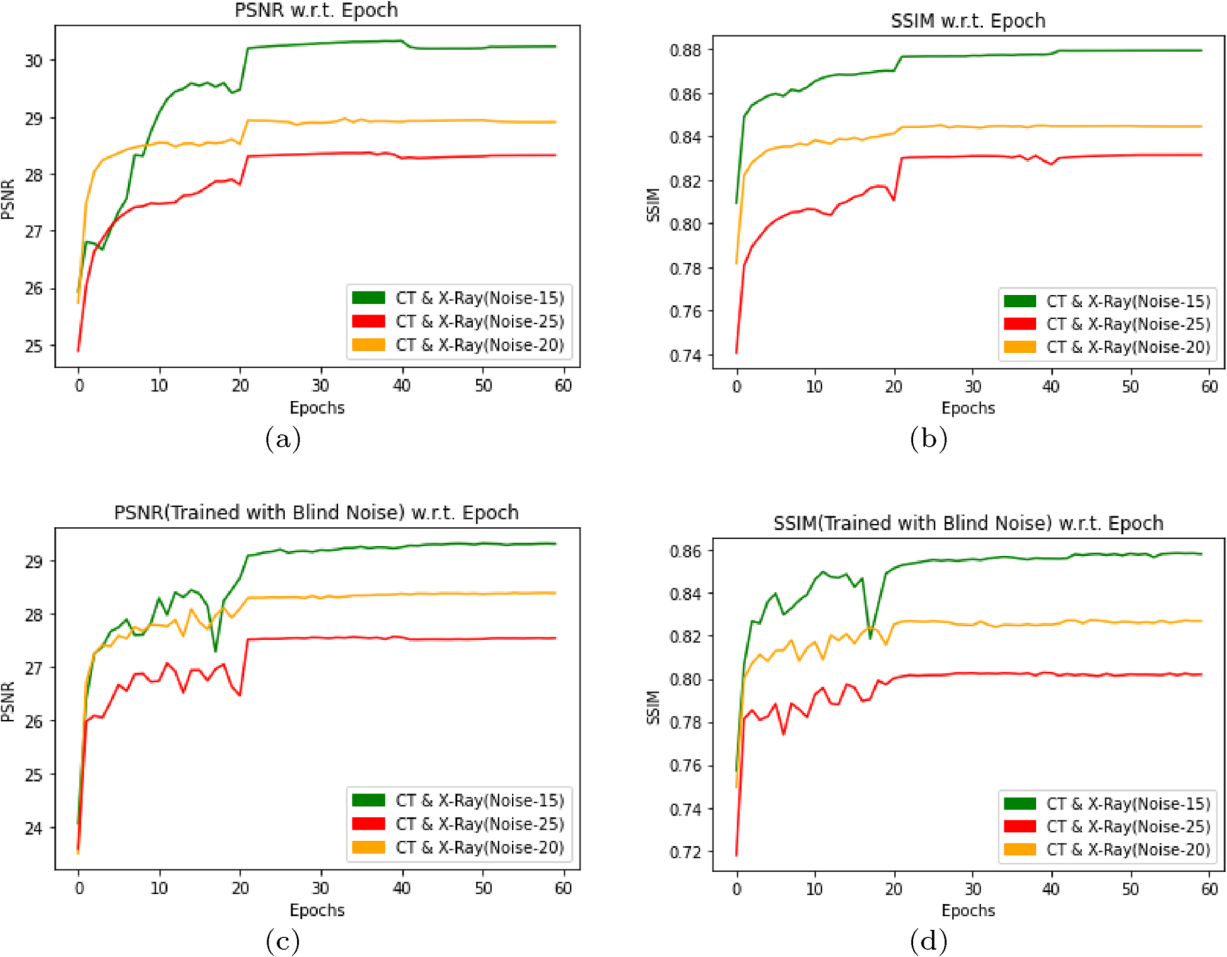
PSNR and SSIM w.r.t. Epoch by using DMF-Net without augmentation and with specific noise in (**a**, **b**) and blind noise in (**c**, **d**) for noise level 15,20 and 25

**Fig. 8:**
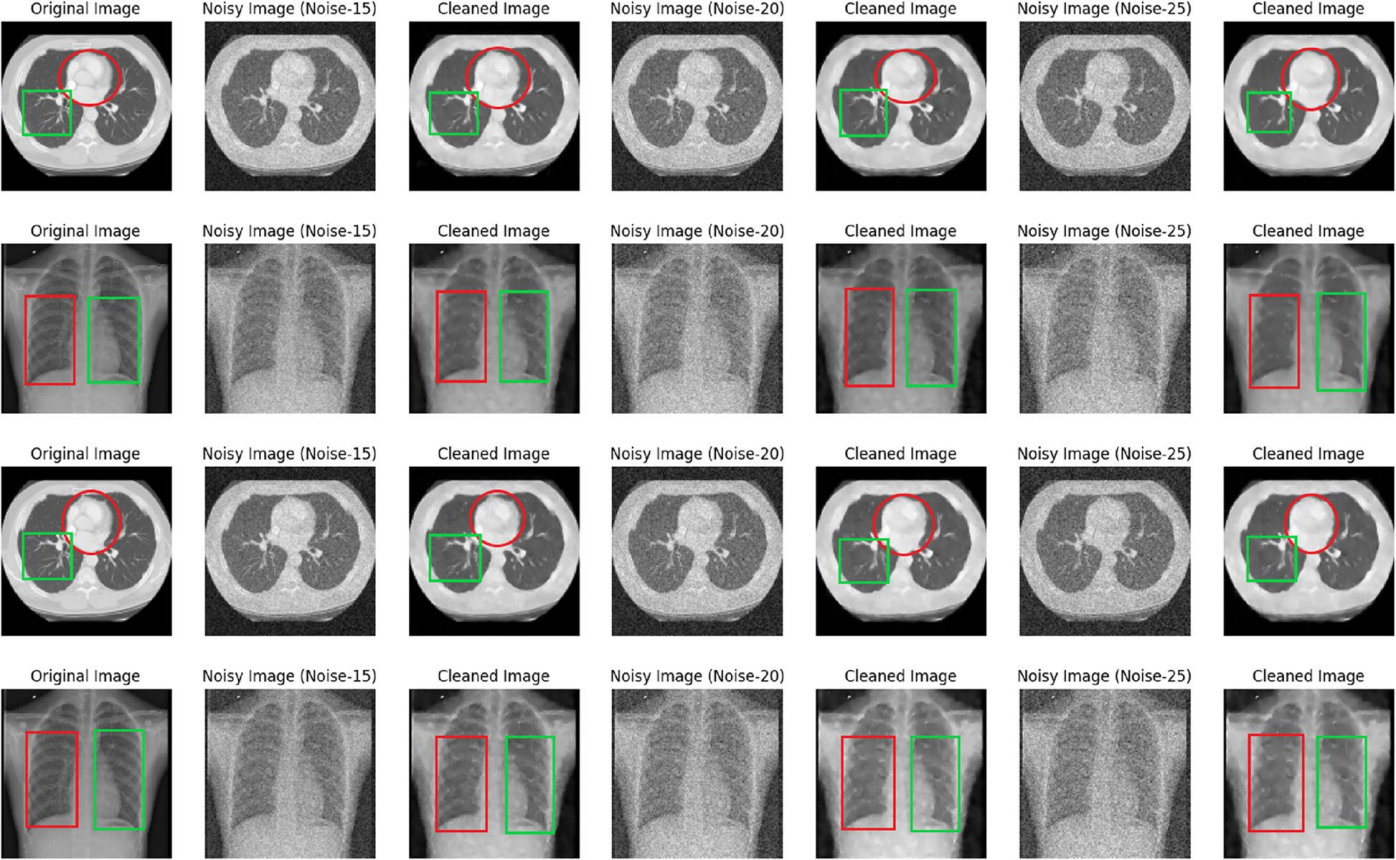
Original CT and X-Ray image with noisy($N_{sp}$=15,20,25 and $N_{bl}$=0 to 55) and denoised or cleaned image by DMF-Net where same images of Figs. 4 and 6 are shown with specific noise and blind noise

In this section, DMF-Net trained with mixed data set of $128\times 128$ X-Ray image and CT images. The data set contains 1292 X-Ray image files and 1365 CT image files. The PSNR and SSIM has been observed for the model after training with specific noise($N_{sp}$) and blind noise($N_{bl}$). The model trained only without augmentation for 60 epochs and it is tested with 553 X-Ray and CT-images with $N_{sp}$=15, 20 and 25 resulting average PSNR and SSIM with [(30.24, 0.8794), (28.91,0.8445) and (28.32,0.8314)] respectively as shown in Fig. 7(a) and (b). Also trained with blind noise $N_{bl}$=0 to 55 resulting average PSNR and SSIM with [(29.30, 0.8582), (28.37,0.8270) and (27.53,0.8020)] respectively as shown in Fig. 7(c) and (d). The input CT and X-Ray images with output denoised images has been shown in Fig. 8.

### State of art comparision

The proposed model DMF-Net compared with other de-noising models like BM3D [20], WNNM [26],NLM [27], BLS-GSM [28], RED-CNN [29], autoencoder [30], CNN-Wavelets [22], non-local means [31], frequency domain FFT [32], adaptive tensor with PCA [33], coefficient driven variation [46], phase preserving [34], optimal weight [35] and wavelet with sparse [30] . The experimental results on specified and blind noise levels are shown in Table 3 to verify the suggested model’s performance. The above methods are evaluated with three different noise levels of 15, 20 and 25. At higher noise levels, our model outperformed the BM3D, WNNM, NLM, and BLS-GSM techniques in terms of PSNR and SSIM. Also our model performance has been evaluated with different medical images like X-Ray and CT as in Table 2, it is found that the performance is better in different two different types of images taken separately or mixed with blind noise and in specific noise. All the result has been compared with different existing models and our proposed model has shown the improved performance over all these.

**Table 2:** Using CT and X-Ray images individually and combined, PSNR and SSIM calculated at different noise levels, i.e. specific noise ($N_{sp}$) and blind noise($N_{bl}$)

Trained	Image	Noise=15		Noise=20		Noise=25	
		PSNR	SSIM	PSNR	SSIM	PSNR	SSIM
**Specific Noise**	CT(No Aug)	29.33	0.8585	27.99	0.8230	26.95	0.7981
	CT(Aug)	29.06	0.8592	27.95	0.8316	26.62	0.7902
	X-Ray(No Aug)	30.94	0.8848	29.72	0.8560	28.72	0.8282
	X-Ray(Aug)	**31.03**	**0.8896**	**29.95**	**0.8637**	**29.19**	**0.8442**
	CT+X-Ray	30.24	0.8794	28.91	0.8445	28.32	0.8314
**Blind Noise**	CT(No Aug)	26.66	0.7973	27.10	0.8082	24.96	0.7444
	CT(Aug)	28.23	0.8294	27.11	0.7934	26.57	0.7787
	X-Ray(No Aug)	28.59	0.8445	27.72	0.8185	26.94	0.7926
	X-Ray(Aug)	28.92	0.8525	28.01	0.8240	27.19	0.7954
	CT+X-Ray	**29.30**	**0.8582**	**28.37**	**0.8270**	**27.53**	**0.8020**

**Fig. 9:**
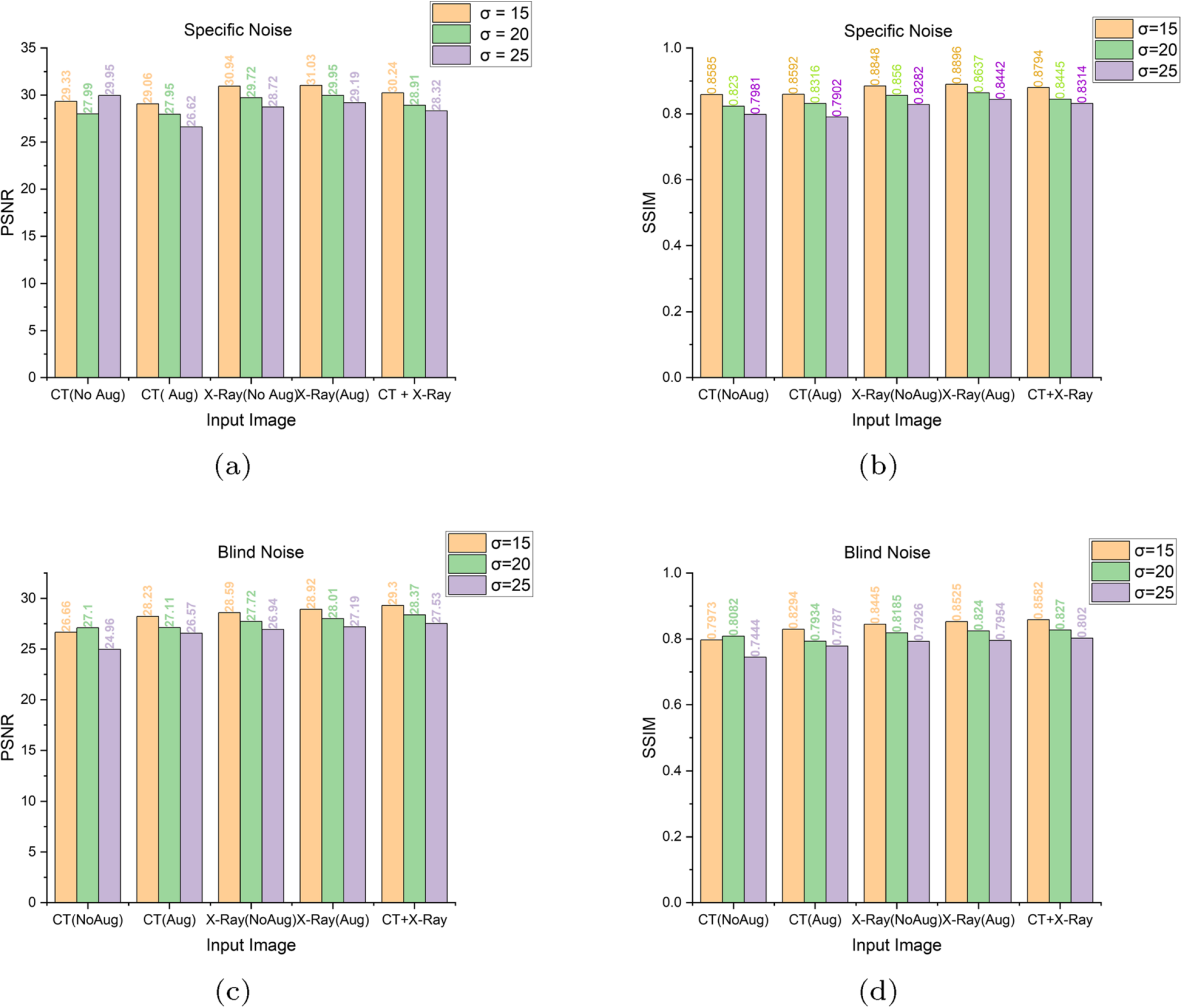
PSNR and SSIM w.r.t. input CT and X-Ray, where (**a**, **b**) shows result when the model trained and validated with specific noise($\sigma$=15,20 and 25) and (**c**, **d**) shows result when the model trained with blind noise and validated with noise level($\sigma$=15,20 and 25)

**Table 3:** Multiple methods were used to acquire PSNR and SSIM measurements at varying CT and X-Ray noise levels (blind and specified noise levels); the best results are highlighted in bold

Trained	Methods	Noise($\sigma$)=15		Noise($\sigma$)=20		Noise($\sigma$)=25	
		PSNR	SSIM	PSNR	SSIM	PSNR	SSIM
**Specific Noise**	BM3D [20]	21.99	0.3778	18.63	0.2671	16.08	0.1381
	WNNM [26]	21.92	0.3669	18.23	0.2356	16.09	0.1345
	NLM [27]	24.61	0.7290	22.32	0.6431	20.29	0.5430
	BLS-GSM [28]	30.60	0.8581	29.09	0.8146	27.99	0.7766
	RED-CNN [29]	30.46	0.8878	23.98	0.7109	18.19	0.6691
	Autoencoder(ANN) [30]	30.30	0.8091	22.11	0.7209	21.34	0.6788
	CNN and Wavelets [22]	30.21	0.8912	23.12	0.7217	21.87	0.6823
	Non-local means [31]	28.71	0.8371	23.28	0.7154	18.76	0.6544
**Blind Noise**	Freq. Domain FFT [32]	29.85	0.8822	22.91	0.6992	17.21	0.6141
	Adapt. Tensor, PCA [33]	29.46	0.8731	22.74	0.6963	18.31	0.6129
	Coeff. Driven Variation [46]	28.75	0.8563	22.41	0.6871	17.78	0.5821
	Phase-preserving [34]	28.13	0.8429	22.09	0.6776	17.13	0.6011
	Optimal Weight [35]	30.17	0.8879	23.08	0.7050	-	-
	Wavelet and Sparse [47]	30.23	0.8078	22.52	0.7053	-	-
	DMF-Net(proposed)	**31.03**	**0.8896**	**29.95**	**0.8637**	**29.19**	**0.8442**
	DMF-Net(proposed)	29.30	**0.8582**	28.37	**0.8270**	27.53	**0.8020**

## Conclusion

In this work, we developed DMF-Net with a strategy of training the network with for de-noising CT and X-Ray images. Here the network is designed with dilated feature extraction block (DEFB), cascaded feature block(CFB) and prominent feature extraction block(PFEB) block. The model consist of total 26 convolutional block where 18 convolution+BN+ReLU(green box), 3 dilated convolution+ReLU (violet box), 4 convolution(orange box), 1 convolution+ ReLU (navy blue box) and Tanh function. The features of the last layers in the cascaded feature block are added with the intention to consider the low, mid and high-level features for evaluating the net noise. The clean image is extracted from the prominent feature extraction block after several convolutions. Here the model is trained and tested separately for CT images, X-Ray images and mixed images for specific and blind noise. The Fig. 9 shows result when the model trained and validated with specific noise($\sigma$=15,20 and 25)(Fig. 9(a), (b)) and Fig. 9(c), (d) shows result when the model trained with blind noise and validated with noise level($\sigma$=15,20 and 25). The network was trained for 60 epochs with just a dynamic decaying learning rate, and it was noticed that in the case of blind noise, mixed images perform better than individual images with and without augmentation and that a good result can also be found in specific noise when using peak signal-noise ratio (PSNR) and structural similarity index measurement (SSIM) as evaluation metrics. As a consequence, the proposed model can learn features taken from mixed images and satisfactorily denoise both CT and X-ray images. Situations where sufficient data is not readily available in advance for training, or when existing data must be modified to account for novel patterns, are ideal applications for real-time machine learning. The preserved state of the trained DMF-Net can be deployed there as an event-driven model to provide real-time generation of clean images with levels of precision.

## Data Availability

The datasets generated and/or analysed during the current study are available in the Kaggle repository, [https://www.kaggle.com/datasets/plameneduardo/sarscov2-ctscan-dataset][https://www.kaggle.com/datasets/andrewmvd/pediatric-pneumonia-chest-xray].
